# Study on the acoustic emission characteristics and damage mechanism of power batteries under electrical load

**DOI:** 10.1371/journal.pone.0333277

**Published:** 2025-09-29

**Authors:** Peijian Jin, Xinwan Xu, Jinrong Xu, Shuo Yang, Wei Yan, Hui Miao, Shimei Sun

**Affiliations:** 1 School of Emergency Science and Engineering, Jilin Jianzhu University, Changchun, Jilin, China; 2 Jilin Provincial Key Laboratory of Fire Risk Prevention and Emergency Rescue for Building, ChangChun, China; 3 Gotion High-Tech Co, Ltd., Hefei, Anhui, China; 4 Ji Lin Sinopoly New Energy Technology Co, Ltd., Liaoyuan, Jilin, China; Monash University, AUSTRALIA

## Abstract

With the rapid development of new energy vehicles and renewable energy storage systems, the safety and reliability of lithium-ion batteries have garnered significant attention. Therefore, it is crucial to study the aging and damage mechanisms of these batteries, explore effective damage monitoring and characterization methods, and improve their safety. This paper constructs an acoustic emission monitoring platform to assess the aging and damage of lithium-ion batteries during charging and discharging processes. It examines the damage mechanisms of both new and aged batteries under various electrical load conditions. All battery groups produced pulse-type acoustic emission signals during the charging and discharging processes at 1C and 0.5C. The acoustic emission waveforms exhibited a dual-peak characteristic throughout the entire charging and discharging cycles. We observed a pattern in which the time intervals between the waveforms decreased rapidly at first and then stabilized. Based on the frequency characteristics analysis of dual waveforms in acoustic emission signals, we propose a hypothesis regarding the formation mechanism of time differences caused by the propagation of acoustic emission signals from the same source through different media to the sensor. The research findings demonstrate the feasibility of non-destructive acoustic emission monitoring during the charging and discharging processes of lithium-ion batteries, offering new technical support for assessing their health status.

## Introduction

Over the past few decades, lithium-ion batteries have been widely used in energy storage devices, electric vehicles, electronic equipment, and other fields due to their high energy density, minimal environmental pollution, and long cycle life [1]. Despite continuous advancements in power battery technology, safety issues still occasionally arise. Failures in power batteries can not only lead to significant economic losses but also pose a threat to people’s lives. The State of Health (SOH) is an important performance metric for lithium-ion batteries. From a capacity perspective, SOH refers to the percentage of the current capacity relative to the initial rated capacity. According to the IEEE 1188–1996 standard, power batteries should be replaced when their SOH drops to 80%. Therefore, to ensure battery safety and to detect potential hazards in lithium-ion batteries early on, it is necessary to conduct status monitoring of the SOH of lithium-ion batteries [2–4].

Lithium-ion battery health status detection methods mainly include experimental methods [5], model-based methods [6–8], and data-driven methods [9]. In experimental methods, instruments and equipment are used to inspect batteries, which are employed to acquire relevant parameters and analyze battery failures [10]. L. Zhang [11] and colleagues utilized X-ray diffraction (XRD) and X-ray absorption spectroscopy (XAS) to study the degradation and aging of lithium cobalt oxide batteries, providing theoretical support for the health status detection of lithium-ion batteries. T. Goh [12] and colleagues established a method for estimating the State of Health (SOH) of lithium-ion batteries online based on the peak positions and values of the differential capacity curves derived from second-order differential voltage curves. Electrochemical mechanism models and equivalent circuit models are the two main approaches in model-based methods. Electrochemical mechanism models have higher accuracy than equivalent circuit models and can predict external physical quantities such as battery current and voltage; however, equivalent circuit models are simpler in computational complexity than electrochemical models but cannot fully reflect the dynamic characteristics of lithium batteries. T.R. Ashwin [13] and colleagues combined the mechanism model of lithium-ion batteries with the theory of how changes in porosity affect battery performance, hypothesizing the formation process of the solid electrolyte interphase (SEI) film under different charging cutoff voltages and rates, constructing a capacity decay model for lithium-ion batteries as the number of cycles increases. T. Feng [14] used sliding average noise to represent the battery cycle structure with a first-order RC equivalent model, and by applying the extended least squares method to real-time discrimination of equivalent circuit model parameters, real-time estimation of lithium-ion battery power conditions was achieved, yielding promising results. E. Sarasketa-Zabala [15] and colleagues studied the effects of lithium-ion battery State of Charge (SOC), depth of discharge, temperature, rate, and total Ah on battery aging, and based on these research results, they established an empirical model for lithium-ion battery capacity decay. M. Petit [16] and colleagues used electrothermal models to analyze lithium-ion batteries, constructing an empirical model for how battery capacity changes with cycle count, while also analyzing the effects of storage and cycling on lithium-ion batteries, which can be applied to other aspects of lithium-ion battery analysis. Data-driven methods process signal data based on battery monitoring data, target signal characteristics, support vector regression, neural networks, fuzzy logic, Bayesian theory, and other methods to determine whether the battery has failed [17,18]. Y. Wang [19] and colleagues estimated the SOC state of lithium-ion batteries using a data-driven approach, employing a moving window neural network structure to determine the energy state of lithium-ion batteries, resulting in a probabilistic joint estimator for lithium-ion battery SOC and energy state. G. W. You [20] and colleagues utilized neural network algorithms to analyze the historical distribution density of various parameters of lithium-ion batteries, such as voltage, current, and temperature data, which was used for estimating a data-driven framework for lithium-ion battery SOH.

Acoustic emission technology has been widely applied in the field of non-destructive testing and damage monitoring for materials such as metals, rocks, composite materials, and magnetic materials [21–24]. Carlos [25] applied acoustic emission technology to the fault detection and diagnosis of wind turbine blades, proposing a new localization method that can accurately detect the location of simulated defects using acoustic emission signals. Since the 1990s, scientists have been using acoustic emission technology to explore the electrochemical behavior of batteries. There are mainly two methods in the analysis of acoustic emission signals: parameter analysis and waveform analysis. The parameter analysis method focuses on recording several key characteristic parameters of the acoustic emission signal, including ring count, event count, duration, threshold, peak, kurtosis, etc., but it does not involve recording the specific waveform of the acoustic emission [26]. In contrast, waveform analysis meticulously records the waveform of each event and obtains more information about the battery status through in-depth analysis of the waveform of the acoustic emission signal in the time domain or frequency domain. Nan Zhou [27] established an experimental platform capable of detecting external short circuits of batteries and acquiring acoustic, electrode, and temperature responses. By selecting appropriate acoustic characteristic parameters in the time and frequency domains, the acoustic response characteristics of different initial battery charge states were analyzed. Nejra Beganovic [28] proposed a remaining useful life/health status estimation model based on acoustic emission technology, which does not require a large number of difficult-to-obtain parameters indirectly related to remaining life/health status. A. Tranchot [29], Pallab Barai [30], and Simon Schweidler [31,32] studied the chemomechanical degradation behavior of lithium-ion batteries from different perspectives based on acoustic emission technology. Kai Zhang [33], after an in-depth analysis of stress wave signals in lithium-ion batteries, successfully identified two key stress wave signal characteristics that reflect the health status of lithium-ion batteries. Kaifeng Wang [1] and colleagues, through waveform analysis of acoustic emission signals, obtained two acoustic emission characteristic parameters: waveform time interval and signal time interval, and proved that they can be used to identify constant current or constant voltage charging modes. Yin Jianxiang [34] deeply explored the correlation between the characteristic parameters of acoustic emission signals and the number of battery cycles and verified the effectiveness of these parameters as an assessment of the health status of lithium-ion batteries.

Scholars’ research on battery safety and acoustic emission technology in batteries varies slightly. Most research on battery safety focuses on state prediction and accident warning, analyzing the overall characteristics of acoustic emission signals. However, there has been no reasonable speculation or validation regarding the generation mechanism of the dual-peak waveform signals during the charging and discharging processes of lithium-ion batteries. The generation mechanism and variation patterns of the dual-peak waveform signals during battery charging and discharging still require further study. This paper aims to explore the formation mechanism of dual-peak acoustic emission signals in lithium-ion batteries by studying the characteristics of acoustic emission signals during the charging and discharging processes of new and aged lithium-ion batteries at different rates. The study has validated the feasibility of non-destructive monitoring of lithium-ion batteries using acoustic emission during charging and discharging, with the expectation of providing new technical support for assessing lithium-ion battery health status.

## Experiment design

### Experimental scheme

#### Experimental materials.

**Fig 1:**
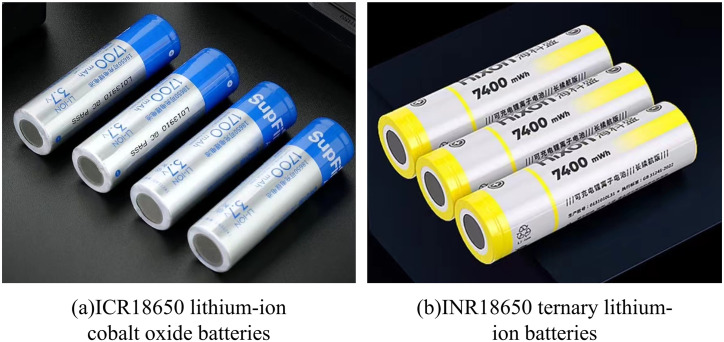
Experimental lithium-ion batteries.

Experimental materials were selected from the more commonly used ICR18650 cobalt-acid lithium-ion batteries (cathode LiCoO_2_, anode C, as shown in Fig 1(a)) and INR18650 ternary lithium-ion batteries (cathode nickel-cobalt-aluminum acid lithium, anode C, as shown in Fig 1(b)) for battery cycle aging experiments and acoustic emission monitoring experiments.

The dimensions of both types of batteries selected for this paper are 65 mm in height and 18 mm in diameter. Since the two batteries have the same exterior shape, the sensors and clamp positions used in the cycle aging experiments and acoustic emission experiments are consistent, ensuring the uniformity of the experimental process and reducing the possibility of experimental errors due to unnecessary factors. The specific product parameters of the batteries used in the experiment are shown in Table 1.

**Table 1:** Battery parameters.

Model	Rated capacity	Nominal voltage	End-of-discharge voltage	Rated charging voltage
ICR18650	1700mAh	3.7V	2.75V	4.2V
INR18650	2000mAh	3.7V	3V	4.2V

From the table, it can be seen that the two lithium-ion batteries have different nominal capacities, both with a nominal voltage of 3.7V and a rated charging voltage of 4.2V. The discharge cut-off voltages for the ICR18650 and INR18650 are 2.75V and 3V, respectively. This provides data support for the subsequent cycle aging experiment plan and acoustic emission monitoring plan, and it is necessary to consider the impact of product parameter differences in parameter settings.

#### Lithium-ion battery cycle aging test protocol.

When conducting cycle aging tests on lithium-ion batteries, the battery samples are divided into two groups based on the battery model: the first group consists of ICR18650 lithium-ion batteries, and the second group consists of INR18650 lithium-ion batteries. Each group of experiments includes 6 battery samples, all of which are subjected to a charging-discharging cycle test using a constant current charging to constant voltage charging, followed by a switch to constant current discharging mode. The cycling parameters used in the experiment are set based on the extreme cycling parameters of lithium-ion batteries, ensuring that the experimental settings do not exceed the safe operating range of the batteries. The specific experimental parameters are selected and can be found in Table 2.

**Table 2:** The aging parameters of each group of batteries.

Cyclic aging parameters	Charge current	Charge voltage	Discharge current	Number of cycles
Group A	850mA	4.2V	850mA	100
Group B	2000mA	4.2V	2000mA	100

The cycle aging test protocol for batteries is conducted outside of the acoustic emission monitoring period, with the core objective being to design, based on the characteristics of the two types of lithium-ion batteries and their performance in actual application scenarios, multiple sets of lithium-ion batteries that have undergone 100 standard charge-discharge cycles, resulting in consistent cycle counts but varying degrees of aging.

#### Lithium-ion battery acoustic emission test protocol.

**Fig 2:**
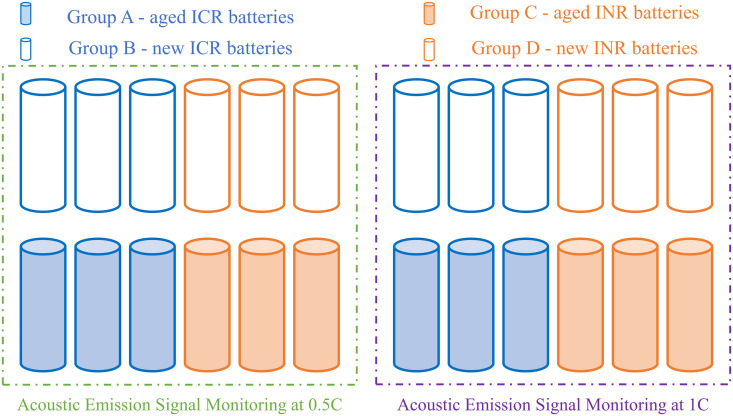
Acoustic emission experimental scheme for lithium-ion batteries.

Based on the age and model of the batteries, the battery samples were divided into four groups for acoustic emission monitoring experiments during the charging and discharging process at 0.5C and 1C rates: Group A and Group B were the aged and non-aged ICR18650 lithium-ion batteries, respectively, while Group C and Group D were the aged and non-aged INR18650 lithium-ion batteries, respectively. Each group used 6 battery samples, with 3 samples used for each rate experiment, and all adopted a charging-discharging mode that switched from constant current charging to constant voltage charging and then to constant current discharging. The specific experimental scheme is shown in Fig 2.

### Experimental platform setup

**Fig 3:**
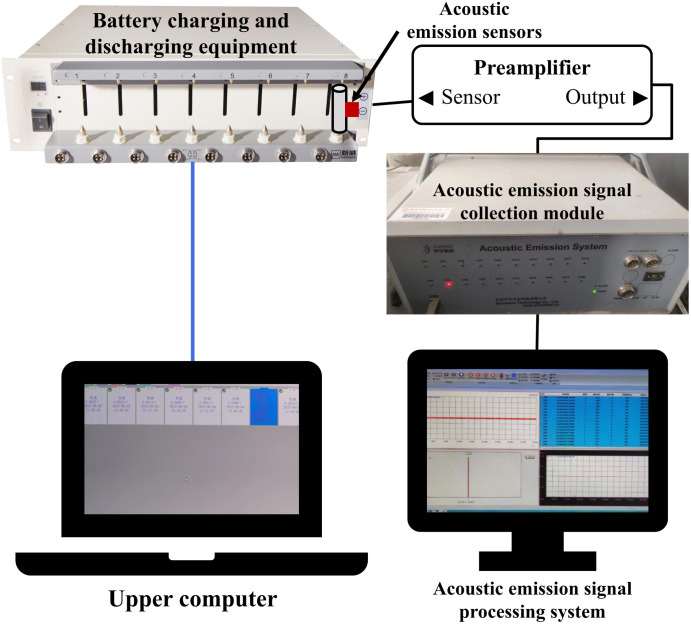
Connection of lithium-ion battery acoustic emission experimental equipment.

The cycling aging experiment of lithium-ion batteries and the acoustic emission experiment is completed on the lithium battery charging and discharging platform, where Neware CT-4008Tn-5V6A battery testing equipment is selected as the battery charging and discharging device. The lithium-ion battery acoustic emission monitoring platform mainly consists of an acoustic emission signal processing system, an acoustic emission signal collection module, a preamplifier, and acoustic emission signal sensors, as shown in Fig 3.

Attach the acoustic emission sensor probe to the lithium-ion battery surface and secure them firmly together. Place the battery on the charging and discharging platform for charging and discharging, and the acoustic emission collection device collects the acoustic emission data and transmits it to the acoustic emission signal processing software on the computer. Save the collected acoustic emission signal data as well as the electrical data. The acoustic emission signal collection module uses the SAEU2S-1016 Full Information Acoustic Emission Signal Analyzer produced by Beijing Sound China Xingye Technology Co., Ltd. To reduce the loss of acoustic emission signals, a layer of HC-98 ultrasonic thickness gauge-specific coupling agent is applied between the sensor and the battery, and insulating tape is used to secure the acoustic emission sensor to the battery. The preamplifier is connected to the sensor; this experiment uses the PAI preamplifier from Qingcheng Acoustic Emission Research Co., Ltd., with an amplification factor of 40dB, a threshold of 45dB, and a sampling rate of 2MSPS. This experiment uses the SR150M sensor for collecting acoustic emission signals, which has a diameter of 19 mm and a thickness of 15 mm; the operating temperature range is −20 ~ 120°C; the frequency range is 60 ~ 400KHz; the sensitivity peak is > 75dB; and the protection level is IP62.

### Experimental results

#### Cycling aging damage experiment.

During the use of lithium-ion batteries, various internal components gradually degrade over time, a phenomenon known as battery aging. Battery aging leads to a decline in battery performance and may also trigger safety issues. Specifically, the main causes of lithium-ion battery aging include the following aspects: Loss of Lithium Inventory (LLI), Loss of Active Material (LAM), and the increase of Ohmic internal resistance [35]. Among them, the reduction of available lithium ions and the loss of electrode active materials are the main reasons for the decrease in the capacity of lithium-ion batteries.

**Fig 4:**
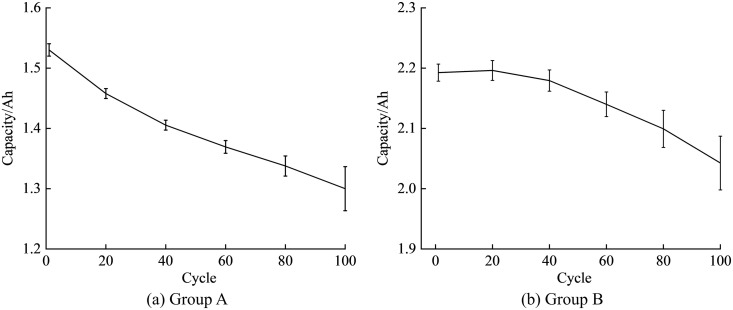
Capacity curve of lithium-ion battery cyclic aging.

The capacity change curves of Group A and Group B batteries after 100 cycles of aging are shown in Fig 4. The initial discharge capacities of the 6 batteries in Group A are 1.5248Ah, 1.5227Ah, 1.5437Ah, 1.5379Ah, 1.5169Ah, and 1.5353Ah, with an average of 1.5302Ah. After 100 cycles of aging, the capacities decreased to 86.6%, 84.3%, 80.1%, 85.6%, 88.3%, and 85.0% of their initial capacities, with an average of 85.0%. The initial discharge capacities of the batteries in Group B are 2.1946Ah, 2.2057Ah, 2.1732Ah, 2.1776Ah, 2.1980Ah, and 2.2065Ah, with an average of 2.1926Ah. After 100 cycles of aging, the capacities decreased to 92.6%, 94.3%, 92.9%, 90.8%, 93.0%, and 95.3% of their initial capacities, with an average of 93.2%. Therefore, the remaining capacity of Group A batteries is approaching the threshold for end-of-life, and compared to Group A, Group B batteries have a considerable margin from 80% SOH. Due to the differences in performance, capacity, and daily practical use between Group A and Group B lithium-ion batteries, Group A uses a 0.5C rate for charging and discharging in the cycle aging, while Group B uses a 1C rate. Group B batteries show a temporary increase in capacity at the beginning of the cycle aging, which may be related to electrode activation. After the material has been fully charged and discharged a few times, it becomes fully activated, and there may be a phenomenon of battery capacity increase.

**Fig 5:**
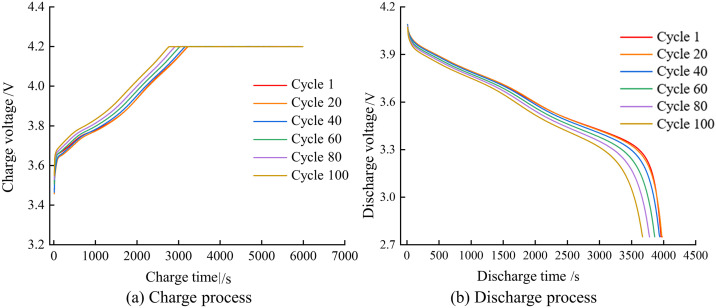
Battery voltage changes during discharge and charge.

The pattern of voltage change during charging and discharging is consistent across batteries with different performance capacities. Taking Group B batteries as an example, Fig 5 illustrates the relationship between voltage and time during the repeated charging and discharging cycles of Group B batteries. As the batteries undergo continuous charging and discharging, the rate of voltage decline during discharge noticeably accelerates, indicating a capacity fade of the battery.

During the constant current-constant voltage (CC-CV) charging process of a battery, as the number of cycles increases, lithium is consumed due to chemical reactions between the electrolyte and electrode materials, leading to an increase in the internal resistance and capacity decay of lithium-ion batteries [35]. As the battery ages, its internal resistance increases, which causes the battery voltage to rise more quickly during the constant current charging phase, thus reducing the time required to reach the preset cut-off voltage. Due to the increased internal resistance, the voltage rise during the constant current charging phase accelerates, resulting in a shortened time for this phase. After the battery ages, the current slope during the constant current charging phase increases, which is because the increased internal resistance leads to a faster voltage rise.

#### Charging and discharging acoustic emission experiment.

Taking one of the experimental batteries in this paper, the ICR18650 lithium-ion battery, as an example, the positive electrode material is LiCoO2, and the negative electrode material is graphite. During the battery cycling process, taking the discharge reaction at the electrode as an example, the reaction equations are shown in Equations (1) and (2):


$$\text{Cathode}:\;{\text{Li}}_{\text{1}-\text{X}}\text{Co}{\text{O}}_{\text{2}}+\text{xL}{\text{i}}^{+}+\text{x}{\text{e}}^{-}\to \text{LiCo}{\text{O}}_{\text{2}}$$ (1)

$$\text{Anode}:\text{L}{\text{i}}_{\text{X}}{\text{C}}_{\text{6}}\to \text{6C}+\text{x L}{\text{i}}^{+}+\text{x }e^{-}\;$$ (2)

The generation of acoustic emission signals is due to the rapid release of energy from local sources within the material, which triggers transient elastic waves. In conjunction with the study of the aging mechanism of lithium-ion batteries, it can be observed that the volume expansion of lithium-ion batteries is a reversible change. The appearance of electrode expansion is always accompanied by the release of energy, and the acoustic emission signals generated in this state exhibit a sudden decay waveform.

**Fig 6:**
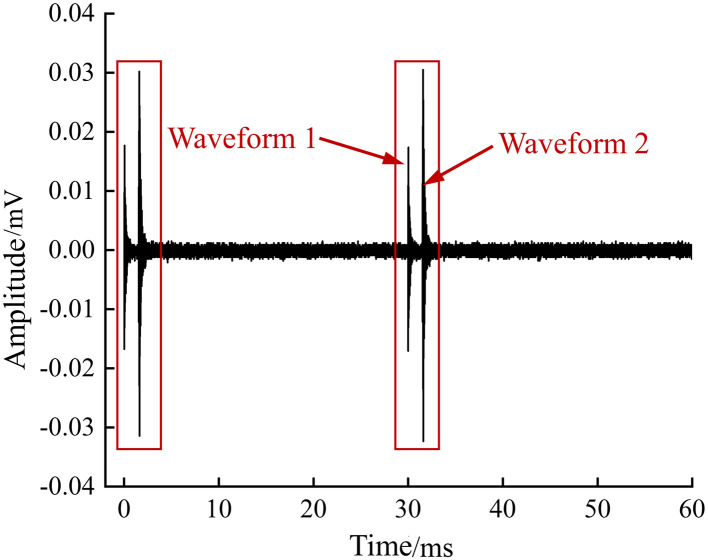
Burst acoustic emission signal recorded in continuous acquisition mode.

This study first collected acoustic emission signal waveforms using a continuous acquisition method with a sampling rate of 1MHz. It was found that in the waveform pages of the acoustic emission signals, there often appears a sudden type of acoustic emission signal composed of two waveforms, as shown in Fig 6. The observation results of this sudden type of acoustic emission signal indicate that some instantaneous physical or chemical changes may have occurred inside the battery.

**Fig 7:**
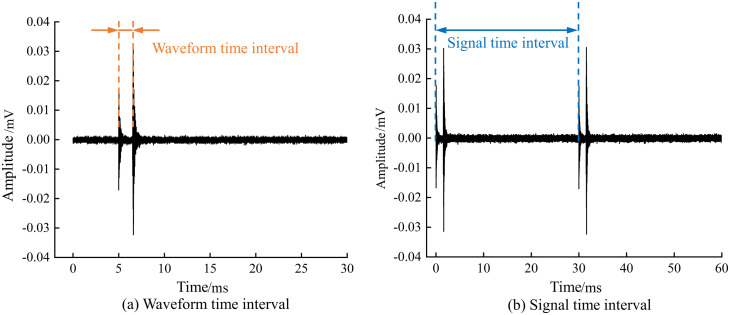
Schematic diagram of two time intervals.

Fig 6 shows two sudden AE waveforms recorded on the same page. This paper designates the first recorded waveform of the acoustic emission signal as Waveform 1 and the second recorded waveform as Waveform 2. By using a threshold-triggered acquisition mode, the system begins to record data when the acoustic emission signal exceeds the set threshold of 40dB, capturing and saving the sudden type of acoustic emission signals that include two waveforms as shown in Fig 6. In the experiment, the post-duration sampling value is set to 5000μs, ensuring the completeness of the signal. Two parameters are defined during the analysis process: waveform time interval and signal time interval. The waveform time interval refers to the time difference between the sampling points where two waveforms in the same event first cross the threshold, while the signal time interval is the time difference between the sampling points where two independent acoustic emission signals first cross the threshold, with the latter used to characterize the generation rate of double-wave acoustic emission signals. Fig 7 provides an illustrative diagram of the waveform time interval and signal time interval.

Groups A, B, C, and D all exhibit a characteristic change in acoustic emission waveform signals during the 0.5C and 1C charging processes: initially, all waveforms are double-waves, then single-waves and double-waves alternate in the middle period, and finally, all are single-waves in the later stage. Among them, the 1C charging process of Group B batteries, the 0.5C charging process of Group D batteries, and the 1C charging process of Group D batteries have significant environmental noise in the acoustic emission waveforms, but it does not affect the observation and analysis of the waveform characteristics. Since the amplitude of Waveform 1 in the double-wave signals during the charging process is slightly smaller than that of Waveform 2, and as charging progresses, the electrical behavior becomes increasingly weak, the amplitudes of the double-wave signals in the charging process are gradually decreasing until the amplitude of Waveform 1 is less than the minimum amplitude set in the threshold acquisition mode, and the single-waves in the later stage of the charging process are all Waveform 2. The acoustic emission waveform signals during the 0.5C and 1C discharging processes for Groups A, B, C, and D are double-waves from beginning to end, and the maximum amplitude of the double-wave signals during the discharging process fluctuates within a certain range without a clear overall increasing or decreasing trend.

**Fig 8:**
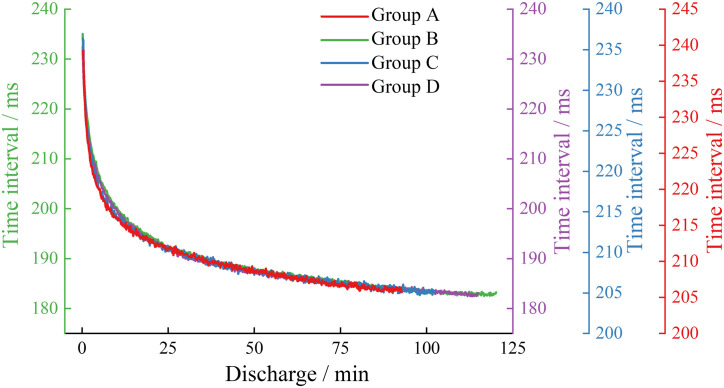
Signal time interval in the 0.5C discharge stage of each group.

**Fig 9:**
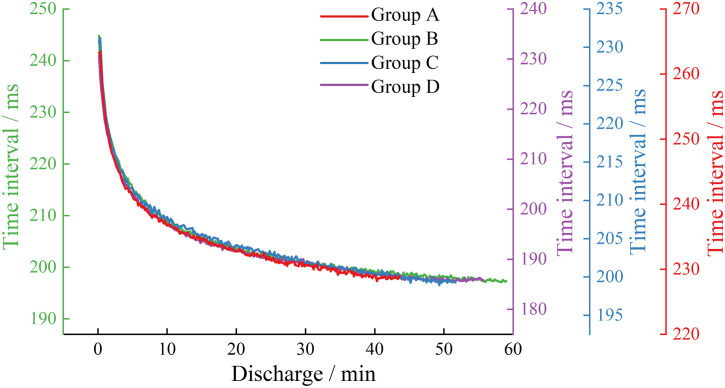
Signal time interval in the 1C discharge stage of each group.

During the charging phase, the double waveform in the acoustic emission waveform of each battery group gradually becomes a single waveform, possibly because the amplitude of the first waveform gradually decays to less than the set threshold, so only the characteristics of the acoustic emission signals during the discharging phase for each group will be analyzed subsequently. As shown in Fig 8, Fig 8 illustrates the signal time intervals of the acoustic emission signals during the discharging phase at a 0.5C rate for each group of batteries. As shown in Fig 9, Fig 9 illustrates the signal time intervals of the acoustic emission signals during the discharging phase at a 1C rate for each group of batteries. From Figs 8 and 9, it can be seen that the signal time intervals for each group of batteries both exhibit two stages: a rapid initial decrease followed by a stable decrease.

At the 0.5C rate, the signal time intervals during the discharge phase of the high-capacity lithium-ion batteries in Groups B and D are shorter than those of the low-capacity lithium-ion batteries in Groups A and C at the same moments, indicating that the reactions during the discharge phase of Groups B and D batteries are more intense than those of Groups A and C. The signal time intervals for the new batteries in Groups C and D are shorter than those of the aged and damaged batteries in Groups A and B, suggesting that the reactions in new batteries occur at a higher frequency than in aged and damaged batteries. At the 1C rate, when comparing the signal time intervals during the discharge phase of the same type of lithium-ion batteries, the new batteries in Groups C and D have shorter intervals than the aged batteries in Groups A and B, which better reflects the reduction in electrode material activity due to cycle aging damage in lithium-ion batteries.

**Fig 10:**
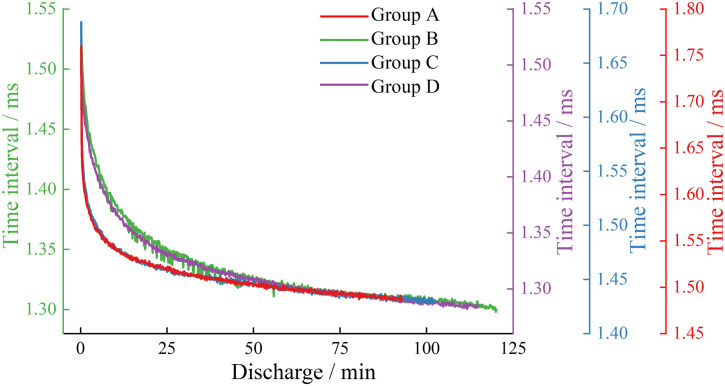
Waveform time interval in the 0.5C discharge stage of each group.

**Fig 11:**
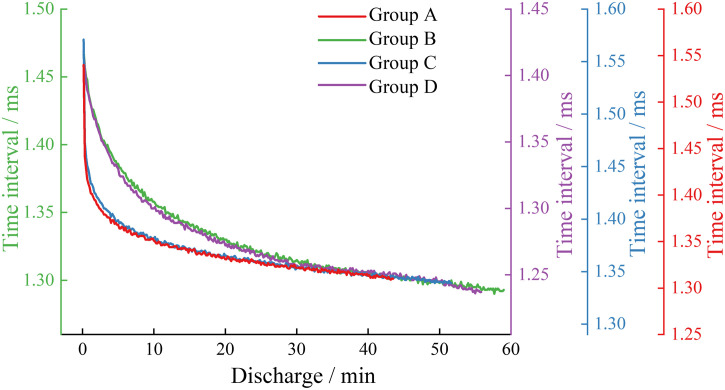
Waveform time interval in the 1C discharge stage of each group.

In the study, the waveform time interval was defined as the time difference between the sampling points where these two types of sudden waveforms first exceed the set threshold. As shown in Fig 10, the acoustic emission signal waveform time intervals were recorded for each group of batteries during discharge at a 0.5C rate, and Fig 11 displays the acoustic emission signal waveform time intervals for each group of batteries during the 1C discharge phase.

From Figs 10 and 11, it can be observed that the waveform time intervals for each group of batteries go through two stages: a rapid initial decrease followed by a stable decrease. At the 0.5C rate, the waveform time intervals during the discharge phase for the high-capacity lithium-ion batteries in Groups B and D are shorter than those for the low-capacity lithium-ion batteries in Groups A and C at the same moments; when comparing the waveform time intervals during the discharge phase for the same type of lithium-ion batteries, the new batteries in Groups C and D have shorter intervals than the aged batteries in Groups A and B. At the 1C rate, the waveform time intervals during the discharge phase for the high-capacity lithium-ion batteries in Groups B and D are also shorter than those for the low-capacity lithium-ion batteries in Groups A and C. Comparing the waveform time intervals at 0.5C and 1C discharge rates, it is evident that the waveform time intervals for all groups of batteries decrease as the discharge rate increases.

The reasons for the above phenomena may be due to the volume changes in the positive and negative materials of lithium-ion batteries during the discharge process as a result of lithium-ion insertion and deinsertion. The negative material experiences a greater volume contraction rate compared to the expansion rate of the positive material due to lithium-ion deinsertion [36–37]. Throughout the battery discharge process, this leads to an increase in the propagation speed of the acoustic emission signals in the solid medium, resulting in a continuous decrease in the double-wave time interval over discharge time. In contrast, aged batteries, relative to new batteries, have a reduced volume change rate in the negative material due to damage, leading to an increase in the double-wave time interval for aged batteries compared to new ones. The discharge rate affects the contraction rate of the negative material by altering the speed of lithium-ion deinsertion, which in turn causes batteries with a higher discharge rate to have a faster volume contraction in the negative material, resulting in a relatively smaller double-wave time interval for batteries with a higher discharge rate under the same conditions.

### Research and discussion

Variational Mode Decomposition (VMD) is a signal processing technique used to decompose signals into multiple modes with different frequencies. VMD is an improvement over Empirical Mode Decomposition (EMD), aiming to address the issues that EMD may encounter when dealing with nonlinear and non-stationary signals. VMD improves upon EMD by introducing variational inference methods, solving some of the problems inherent in EMD, and providing a more effective tool for the field of signal processing.

**Fig 12:**
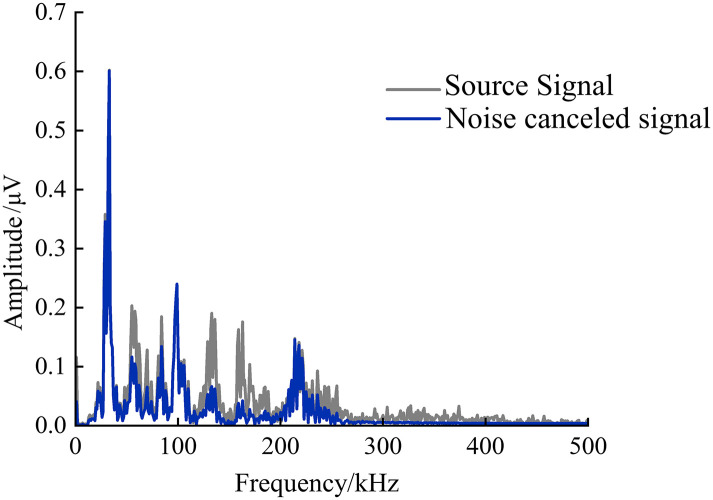
Comparison of acoustic emission signal before and after noise reduction.

Through the VMD algorithm, effective signal components can be extracted from acoustic emission signals, allowing for more accurate identification and analysis of the internal physicochemical processes of batteries. However, before applying the VMD algorithm, it is necessary to initialize some key parameters, among which the number of decompositions and bandwidth parameters significantly affect the final decomposition results. Therefore, it is crucial to reasonably set these parameters before performing VMD decomposition on acoustic emission signals to ensure the accuracy and reliability of the decomposition results. Considering the effect of noise suppression while avoiding modal aliasing and false component issues, the final parameter K is set to 3, and α is set to 2000, meaning the modal components IMF are defined as 3. Fig 12 shows the comparison of lithium-ion battery acoustic emission signals before and after noise reduction when K = 3 and α = 2000.

#### Acoustic emission event peak frequency analysis.

**Fig 13:**
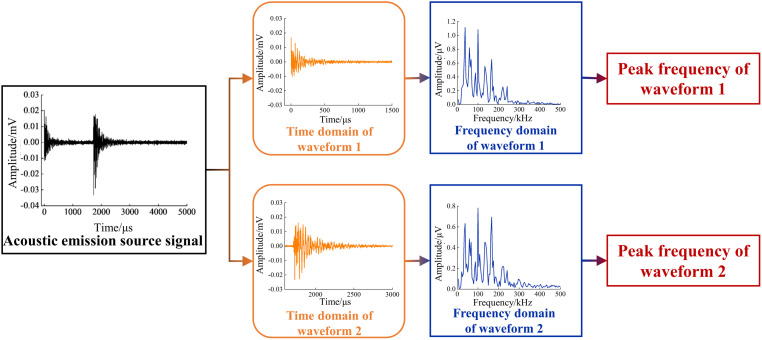
Flowchart of the extraction of the peak frequencies of the two waveforms.

To explore the similarities and differences between waveform 1 and waveform 2 in the frequency domain, as well as their variation patterns, the original single group of dual waveform acoustic emission signals is extracted into individual waveform 1 and waveform 2 acoustic emission signals. Both are then denoised and subjected to FFT to obtain their corresponding frequency domain diagrams. Subsequently, the frequency domain diagrams are analyzed to find the main frequency at which the primary frequency is located. The specific data processing flow is shown in Fig 13.

**Fig 14:**
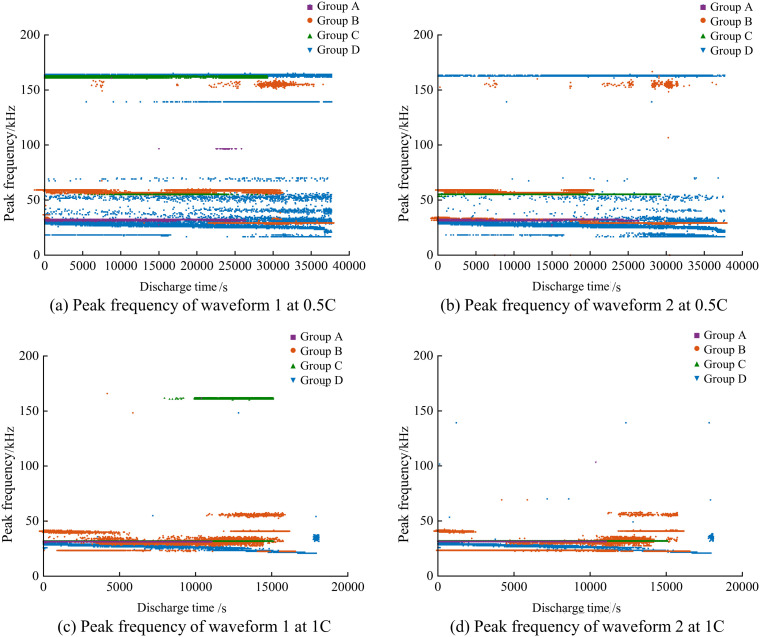
The peak frequency of the two waveforms of the acoustic emission signal of batteries.

For all groups of dual waveforms, the frequency domain analysis was performed individually. The frequency domain distribution of waveform 1 and waveform 2 is similar to that of the single dual waveform acoustic emission signals, both consisting of low-frequency, mid-frequency, and high-frequency segments, with the low frequency being the main frequency. The main frequency of the acoustic emission signals for waveform 1 and waveform 2 is taken as the peak frequency of the corresponding individual acoustic emission events. The statistical results of the peak frequencies of the dual waveform signals for all battery groups are shown in Fig 14.

The peak frequencies are generally distributed within the 0 ~ 200kHz frequency range, with specific manifestations as follows: In Group A batteries, the peak frequencies of waveform 1 and waveform 2 during the discharge process at 0.5C and 1C rates are stable in the 30 ~ 32kHz frequency band. In Group B batteries, the peak frequencies of waveform 1 and waveform 2 during the discharge process at different rates show different patterns of change. Specifically, at the 0.5C rate, waveform 1 and waveform 2 mostly fluctuate between 55 ~ 60kHz, with some fluctuations in the 150 ~ 170kHz range in the middle and later stages, and the trends for waveform 1 and waveform 2 are consistent. At the 1C rate, the peak frequencies of waveform 1 and waveform 2 are in the 30 ~ 60kHz range during the early and middle stages, showing a pattern of decreasing first and then increasing, with the overall trends for waveform 1 and waveform 2 still being consistent. In Group C batteries, the peak frequencies of waveform 1 and waveform 2 during the discharge process at 0.5C and 1C rates are both stable, with waveform 1 and waveform 2 both stable at 55kHz at the 0.5C rate, and both stable at 32kHz at the 1C rate. In Group D batteries, the peak frequencies of waveform 1 and waveform 2 during the discharge process at the 1C rate both show a slow and stable downward trend, mainly fluctuating in the 22 ~ 40kHz frequency band; during the discharge process at the 0.5C rate, the peak frequencies of waveform 1 and waveform 2 both exhibit a high-low frequency distribution pattern, with 80% ~ 90% mainly fluctuating in the 22 ~ 55kHz range, and 10% ~ 20% distributed in the 165kHz range.

At the 0.5C rate, compared to Group C batteries, the peak frequencies of waveform 1 and waveform 2 in the aged Group A batteries are lower than those in the new Group C batteries; compared to Group D batteries, the aged Group B batteries are higher than the new Group D batteries. This may be related to the different aging causes of different types of batteries. At the 1C rate, the peak frequencies of waveform 1 and waveform 2 in Group A and Group C batteries are both close to 32kHz; in Group B and Group D, there is a general phenomenon where the peak frequencies of the aged Group B batteries are higher than those of the new Group D batteries. Additionally, at the 1C rate, the peak frequencies of waveform 1 and waveform 2 in Group B and Group D are more stable overall than at the 0.5C rate. The former’s peak frequency range is 30 ~ 60kHz, while the latter, aside from about 80% within the 30 ~ 60kHz range, has another part distributed in the 150 ~ 170kHz range. Reference [38] confirms that low-frequency (20 ~ 100kHz) acoustic emission signals are produced by gas-generating SEI; high-frequency (100 ~ 300kHz) acoustic emission signals are produced by electrode crack formation. For ICR18650 lithium-ion batteries, the acoustic emission signals during the discharge process are characterized by gas-generating SEI; for INR18650 lithium-ion batteries, the discharge process is mainly characterized by gas-generating SEI, with some phenomena of electrode crack formation as well.

#### Mechanism analysis of acoustic emission waveform characteristics.

Previous scholars have made some speculations and analyses on the generation mechanism of dual waveform acoustic emission signals. For instance, as analyzed in literature [1], the cycling of lithium ions into and out of the crystal structure of active electrode materials changes the shape of the active electrode materials. The lithium intercalation/deintercalation of active electrode particles generates compressive and tensile stresses. These compressive and tensile stresses excite transient elastic waves, which are the acoustic emission signals. Additionally, electrode cracks produce stress waves, which are the pulse-type acoustic emission signals generated when electrode cracks form. The literature speculates that the dual waveforms are two different acoustic emission signals produced by two distinct processes in the lithium intercalation/deintercalation process, the scientific validity of which remains to be debated. The reasons are as follows: The analysis in the previous sections of this study indicates that the frequency distribution and variation of the two acoustic emission signals before and after the dual waveforms are almost identical during a single discharge. We have reason to suspect that there is another possibility for the mechanism of dual waveform acoustic emission signal generation: a single acoustic emission signal is received by the sensor after propagating through different paths. Due to the attenuation during the propagation of acoustic emission and the different velocities of waves, the acoustic emission signals arrive at the sensor with a time difference through two propagation modes, which is the parameter of waveform time interval mentioned earlier in the text.

**Fig 15:**
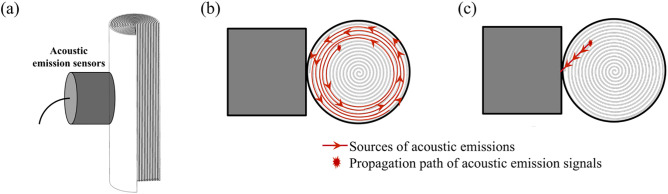
Mechanism of acoustic emission dual waveform signal formation.

Specifically, the formation mechanism of the dual waveform acoustic emission signals is shown in Fig 15. Fig 15(a) is a schematic diagram of the sensor and battery position and structure in the acoustic emission experiment. On one hand, due to the coiled assembly process of cylindrical batteries, the acoustic emission signals propagate from the inside of the battery to the surface along the long and thin electrode foils (Fig 15(b)); on the other hand, the acoustic emission signals generated during the discharge process through chemical reactions are received by the sensor probe through the shortest path from the source location to the battery casing (Fig 15(c)), two routes propagate simultaneously from a single sound source. The velocity of acoustic emission signals in solid materials is between 0.5 and 5 km/s [39,40], and the length of the positive and negative electrode coating is about 600–800 mm. The time difference calculated for the two propagation paths mentioned earlier is between 0.12 and 1.6 ms, and the actual measured time difference is between 1.2 and 1.6 ms. Considering the refraction of sound waves and the attenuation of wave velocity when propagating between different electrode layers [41], it can be proven that the reasoning result is reasonable.

### Conclusions and future work

After analyzing and discussing the results of the acoustic emission experiments on lithium-ion batteries and verifying the generation mechanism of signal characteristics, the following conclusions are mainly drawn:

(1) In the cycling aging damage experiments of lithium-ion batteries, the capacity loss of ICR18650 batteries and INR18650 batteries after 100 cycles of aging is different, with the INR18650 batteries experiencing less capacity loss under high-rate cycling aging than the ICR18650 batteries. During the cycling aging process, the battery voltage rises faster during the constant current charging phase, reducing the time required to reach the cutoff voltage; meanwhile, the battery voltage drops significantly faster during the discharge phase, indicating a decline in battery capacity.
(2) In the acoustic emission experiments, the acoustic emission waveform signals of ICR18650 and INR18650 batteries during the 0.5C and 1C discharge processes are dual waveforms from beginning to end, and the maximum amplitude of the dual waveform signals varies within a certain range throughout the discharge process, showing no clear overall increasing or decreasing trend. Analysis of the waveform time interval and signal time interval indicates that the variation trends of these intervals for all groups of batteries are consistent, both exhibiting two stages of rapid decline followed by stable decline.
(3) The analysis of the dual waveform characteristics of acoustic emission signals during the aging process of lithium-ion batteries shows that the peak frequencies of waveform 1 and waveform 2 change consistently throughout the entire discharge process. It is inferred that the dual waveform signals are formed by a single acoustic emission event generated by a single electrode arriving at the sensor through two different propagation paths, resulting in a time difference. Empirical data calculations have been used to verify the reasonableness of this inference.

In future work, acoustic emission experiments on lithium-ion batteries require more in-depth research and effective validation. On one hand, more experiments related to different external conditions that may affect battery performance should be designed, such as experimental temperature, charge-discharge rates, electrode materials, and battery shape and size, etc.; on the other hand, the verification of the dual waveform characteristics in acoustic emission signals awaits an effective and reasonable means of validation, such as using acoustic emission source localization methods or selecting prismatic batteries for experiments. The localization of the acoustic emission source will be inspired by literature [42] for the next step of work, and the use of prismatic batteries is to observe whether the acoustic emission signals still exhibit time differences and their magnitudes under the same conditions to verify the mechanism of dual waveform generation.

## Supporting information

S1 Data Minimal data set. (ZIP)
